# The Prophylactic Treatment of Placenta Prævia

**Published:** 1878-01

**Authors:** 


### The Prophylactic Treatment of Placenta Prævia.

THE American supplement of the *Obstetrical Journal of Great Britian and Ireland* for May contains a summary of a very interesting article by Dr. T. Gaillard Thomas on the Prophylactic Treatment of Placenta Prævia. The article is published in the *American Practitioner* for May. Dr. Thomas gives the particulars of eleven cases of this unpleasant complication. The results were very satisfactory. Only one of the mothers died, and seven of the eleven children were born alive. Dr. Churchill says that under ordinary management one in three, or thereabouts, of the mothers die, and more than half of the children are lost. The essential feature of Dr. Thomas's practice was the induction of premature labour. In eight of the eleven cases the chief agent in inducing premature labour was the use of Barnes's bags. The placenta was not in all cases to be felt, but where the natural indications of its presence existed Dr. Thomas acted as though he felt it. He did not in all these cases act with haste, or on the first occasion of hæmorrhage, but generally, as soon as he was satisfied that the placenta presented some proof that the further loss of blood was dangerous, he recommended the induction of labour. We believe that there would be a general concurrence of opinion on this subject now. Once satisfied that a case is one of placenta prævia, it is desirable, in the interest both of the mother and of the child, to watch it very closely, and before the patient becomes exsanguine, to proceed to induce labour. Dr. Barnes's judgment on this point will be generally accepted—"If the pregnancy has advanced beyond the seventh month, it will, as a general rule, I think, be wise to proceed to deliver, for the next hæmorrhage may be fatal. We cannot foretell the time or the extent of its occurrence, and when it occurs, all, perhaps, that we shall have the opportunity of doing will be to regret that we did not act when we had the chance." In several of Dr. Thomas's cases, to expedite delivery, version was practised, generally by the bimanual method. In one, Barnes's bag, impinging on the presenting head, caused the child to revolve in the liquor amnii till the breech presented. We notice that in most of the cases before version and delivery were performed the patient was anæsthetised, generally with ether. In the one fatal case the patient had had hæmorrhages during the seventh and eighth month. She came under Dr. Thomas's care at the end of the eighth month. She was carefully watched for Dr. Thomas by Dr. C. S. Ward for a week. "as decided hæmorrhage recurred," premature labor was decided on. The bags were used, and a female catheter was introduced into the uterus. Pains came on in three or four hours, and the patient was delivered of a vigorous male child; but in forty-eight hours puerperal septicæmia came on and proved fatal. Perhaps a better result, even in this case, would have followed earlier induction of labour. One month after, the lady's sister, in the same house, attended by Dr. Sands, after a perfectly natural labour, died of puerperal fever.—*Lancet.*

